# Non-Provitamin A and Provitamin A Carotenoids as Immunomodulators: Recommended Dietary Allowance, Therapeutic Index, or Personalized Nutrition?

**DOI:** 10.1155/2018/4637861

**Published:** 2018-05-09

**Authors:** Elisabetta Toti, C.-Y. Oliver Chen, Maura Palmery, Débora Villaño Valencia, Ilaria Peluso

**Affiliations:** ^1^Research Centre for Food and Nutrition, Council for Agricultural Research and Economics (CREA-AN), Rome, Italy; ^2^Antioxidants Research Laboratory, Jean Mayer USDA Human Nutrition Center on Aging, Tufts University, Boston, MA, USA; ^3^Department of Physiology and Pharmacology, “V. Erspamer”, La Sapienza University of Rome, Rome, Italy; ^4^Universidad Católica San Antonio de Murcia (UCAM), Murcia, Spain

## Abstract

Vegetables and fruits contain non-provitamin A (lycopene, lutein, and zeaxanthin) and provitamin A (*β*-carotene, *β*-cryptoxanthin, and *α*-carotene) carotenoids. Within these compounds, *β*-carotene has been extensively studied for its health benefits, but its supplementation at doses higher than recommended intakes induces adverse effects. *β*-Carotene is converted to retinoic acid (RA), a well-known immunomodulatory molecule. Human interventions suggest that *β*-carotene and lycopene at pharmacological doses affect immune functions after a depletion period of low carotenoid diet. However, these effects appear unrelated to carotenoids and retinol levels in plasma. Local production of RA in the gut-associated lymphoid tissue, as well as the dependency of RA-induced effects on local inflammation, suggests that personalized nutrition/supplementation should be considered in the future. On the other hand, the differential effect of RA and lycopene on transforming growth factor-beta suggests that lycopene supplementation could improve immune functions without increasing risk for cancers. However, such preclinical evidence must be confirmed in human interventions before any recommendations can be made.

## 1. Introduction

Major dietary non-provitamin A (lycopene, lutein, and zeaxanthin) and provitamin A (*β*-carotene, *β*-cryptoxanthin, and *α*-carotene) carotenoids have different biological activities and efficacy, depending on their food content, dietary intake, bioavailability, and bioconversion [1]. The intestine and liver are crucial organs for vitamin A uptake and liver accounts for the majority of retinoid stores [2, 3]. The provitamin A carotenoid, *β*-carotene, is a significant source of vitamin A in the diet. *β*-Carotene ′ oxygenase-1 (BCO1) and *β*-carotene 9′,10′ oxygenase-2 (BCO2) are the two known carotenoid cleavage enzymes in humans [4]. In rats, both BCO1 and BCO2 are highly expressed in the liver and intestine, localized in hepatocytes and mucosal epithelium, and BCO1 is also expressed in hepatic stellate cells [4]. Both enzymes have provitamin A and non-provitamin A as preferential substrates, respectively, and genetic variations of these enzymes have been suggested within the factors affecting carotenoid status in humans [5, 6].

*β*-Carotene is known as an antioxidant, but its prooxidant activity in some conditions accounts for its adverse effects [6]. In particular, *β*-carotene failed to prevent cancer in two large clinical trials: the Alpha-Tocopherol, Beta-Carotene Cancer Prevention Study (ATBC Study; *α*-tocopherol 50 mg and *β*-carotene 20 mg/d) [7] and the Beta-Carotene and Retinol Efficacy Trial (CARET; *β*-carotene 30 mg/d and retinyl palmitate 25,000 IU) [8]. Moreover, *β*-carotene supplementation increased lung cancer risk in smokers [9, 10] and the overall mortality [11, 12]. On the other hand, a safer profile for non-provitamin A carotenoids (up to 20 mg/d for lutein and 75 mg/d for lycopene) has been suggested [13]. Lycopene has been extensively studied [14], and encapsulation has been suggested to improve bioavailability for therapeutic use in many conditions, including immune-mediated diseases [15].

Retinol bound to the retinol-binding protein (RBP) is a source of retinoic acid (RA) [2, 16], and the latter is metabolized by cytochrome P450 26 (CYP26) [3]. After uptake, retinol can be oxidized by ubiquitously expressed alcohol dehydrogenases (ADH) to form retinaldehyde (retinal) which is then metabolized into RA by retinaldehyde/aldehyde dehydrogenases (ALDH) in the liver [3, 17, 18]. ALDH are also expressed in the gut-associated lymphoid tissue (GALT) [3]. Although RA is the major active metabolite affecting the immune system, non-provitamin A carotenoids are active in immune modulation [19]. Furthermore, it has been reported that BCO1 could yield acycloretinal from lycopene [20] and that lycopene-derived BCO2 metabolites could mediate in some circumstance signals similar to that induced by retinoic acid receptor (RAR) ligands [21].

In this review, we aim to discuss the potential role of carotenoids as immunomodulators, on the light of their intake and safety.

## 2. Carotenoid Sources

The major carotenoids present in food products are *β*-carotene, *α*-carotene, *β*-cryptoxanthin, lycopene, lutein, and zeaxanthin [22] (Table 1). With the exception of egg yolk rich in lutein, the main sources of these compounds in human diet are of plant origin; they are widely distributed in the plastids of flowers, leaves, seeds, and roots. Orange, yellow- and green-colored vegetables are the rich sources; lycopene is found abundantly in tomatoes and their related products and is also present in fruits, such as watermelon and pink grapefruit [23]. Citrus fruits, papaya, and peaches contain significant levels of *β*-cryptoxanthin. The xanthophylls lutein and zeaxanthin are mainly found in leafy green vegetables, such as spinach or broccoli [24]. Likewise, an emerging source of carotenoids is the by-products of industry processing of fruits and vegetables [25].

Contents of carotenoids vary widely because their syntheses are greatly influenced by a wide variety of factors, including climate, soil, cultivar, and cultivation [26]. Further, their profile in berries changes with ripening stage, with higher levels of *α*-carotene and lycopene in advanced ripening [27]. In addition to preharvest factors, their contents can be affected by all treatments during postharvest because their highly unsaturated structures with conjugated double bonds make them very susceptible to oxidative reactions and dimerization. For example, cutting of vegetables increases the exposure to oxygen and releases enzymes from the cell vacuoles of plant parenchyma, which further promote their degradation. Excessive exposure to sunlight also decreases the content of carotenoids in harvested products [28]. Degradation of carotenoids can be diminished by storage at low temperatures, protection from light (packaged in dark containers), or package under modified atmospheres. However, the impact of thermal treatments on carotenoids appeared mixed. For example, nonthermally treated tomatoes had higher amounts of carotenoids compared to thermally treated ones and similar results were observed with carrot [29]. However, home culinary techniques, such as boiling in hot water, cause partial degradation and isomerization of both *β*-carotene and lycopene. Current industrial processing techniques as high-pressure treatment tend to preserve or even increase the content of carotenoids [30].

## 3. Dietary Intake, RDA, and Retinol Equivalents

Dietary data on consumption of carotenoids were in the past usually expressed as *β*-carotene, *β*-carotene equivalents, or retinol equivalents, and only more recently, carotenoid food composition databases have been developed. There is a general consensus regarding that the contribution of dietary carotenoids from food sources depends not only on their contents in foods but also on the frequency of their consumptions. Estimated intakes of carotenoids vary widely on individual, regional, and national levels, and significant seasonal variations have also been reported in some countries [31]. Furthermore, assessment of carotenoid intake is a complex matter mainly because of the high variability within and between subjects, the degree of imprecision in data collection, and discrepancies in carotenoid food composition databases, which reflect in different intakes of carotenoids in the literature.

Studies on dietary carotenoids are few, and the main results of one of the few comparative studies are presented in Table 2 [32], where the assessment of carotenoid intakes was carried out by a Food Frequency Questionnaire (FFQ) at the individual level of five countries. It should be noticed that the population in this study was a group in a determined area of each of the five participant countries (ca. per country). Thus, subjects might not necessarily be representative of the overall population although it was assumed that they followed a typical dietary pattern of their countries. Moreover, it should not be ignored that FFQ overestimates carotenoid intake [33], especially of lutein and zeaxanthin when comparing with 3-day food records. Table 2 summarizes carotenoid intake in some countries from the representative literature with a larger sample size. The total carotenoid intakes range between 5.42 and 15.44 mg/d; however, comparisons should be considered with caution since, as shown, sample size and methodology differ between studies.

In a review from Maiani et al. [1], a calculation of the relative contribution of each carotenoid to total carotenoid intake, according to FAO Food Balance Sheet data from several European countries, was performed. Lutein + zeaxanthin and *β*-carotene were those most frequently found in European diet (48% and 33%, respectively, on a total carotenoid intake of 11.8 mg/d). No formal dietary recommendation for carotenoids has yet been established, and the European Food Safety Authority (2006) had decided that the existing evidence was insufficient to establish a recommended dietary allowance (RDA) or adequate intake (AI) for *β*-carotene and other carotenoids [34]. In most European countries, the recommended intake was established based on the assumption that 4.8 mg *β*-carotene is needed to meet the requirement of 800 micrograms of vitamin A (conversion factor 6). In other countries, for example in USA, a conversion factor of 12 for *β*-carotene and 24 for other carotenoids such as *β*-cryptoxanthin was applied [35]. For very complex matrices (i.e., spinach), human studies have revealed an even higher conversion factor for *β*-carotene such as 1 : 21 for a fruit/vegetable mix or 1 : 26 for vegetables [36]. Conclusions of many epidemiological studies revealed that a plasma level of 0.4 *μ*mol/L *β*-carotene should be aimed at in order to benefit from the preventive health potential. This concentration can be achieved with consumption of 2–4 mg/d *β*-carotene [37], far below the supplemented dose used in the ATBC study [7] and the CARET study [8], in which an increased risk of lung cancer was noted in heavy smokers taking high doses (5 to 10 times the dose previously indicated of 2–4 mg/d) of *β*-carotene for long periods.

Consumption of foods rich in *β*-carotene is highly recommended since it is associated with a lower risk of chronic diseases and to ensure the intake of a sufficient amount of antioxidants. Healthy diet, which realistically contains 100–500 g/d of fruit and vegetables, shall contain a high proportion of carotenoid-rich food. On the other hand, proposed intake recommendations for some non-provitamin A carotenoids are 10–20 mg/d for lutein and 5.7–15 mg/d for lycopene [38].

## 4. Bioavailability and Accessibility

Bioavailability of dietary xanthophylls is varied widely between individuals and subject to the influence of many intrinsic and extrinsic factors [51]. Bioavailability is defined as the portion of the ingested nutrients that are absorbed in the small intestine, enter in the circulation, and become available for utilization or storage in organs [52–54]. Before nutrients in foods, beverages, or nutraceuticals are absorbed in the intestine, they must be made themselves ready for the transportation from the chyme in the lumen to enterocytes, a process defined as bioaccessibility. In the case of lipid-soluble carotenoids, ingested carotenoids must be first released from the food matrix, transferred into lipid emulsion, incorporated into the micelles containing pancreatic lipases and bile salts, and then available for transport into enterocytes [54–56]. The micelles act as a polar carrier from the hydrophilic chyme to the mucosal cell surface for the uptake through passive diffusion [57]. The factors influencing carotenoid bioaccessibility and bioavailability can be categorized to carotenoid-related and unrelated groups. The carotenoid-related includes dosage, chemical structure (isomeric forms), and interactions between carotenoids, and the unrelated includes cooking, nutrient composition of co-consumed foods, particle size of digested foods, biometrics of consumers, efficiency of micellarization, and transport from the enterocytes to the lymph system [36, 57–61]. Thus, carotenoid contents in foods may not be well correlated with their bioavailability and the ultimate bioefficacy because of the interference of negative effectors [62]. Among the unrelated factors, presence of dietary fat, heat treatment, and reduced particle size have a noticeable positive effect whereas dietary fibers and proteins have a negative effect [62]. Mechanical processing, including chopping and chewing, help reduce particle size and release carotenoids from chloroplasts and tissue for the bioaccessibility [63–65]. The amounts of naturally occurring lipids are rather low in most carotenoid-rich fruits and vegetables so that 3–5 g of fat intake per day is essential for the optimal absorption of carotenoids [66, 67]. Further, the presence of dietary fats, particularly long-chain fatty acids, for example, oleic acid, is more beneficial for the absorption of nonpolar carotenoids (carotenes) than that of polar ones (xanthophylls) [62, 68–70] because polar carotenoids can be more easily transferred from emulsified lipid to micelles [71]. Dietary fibers, the principle components of plant foods, compromise carotenoid release from food matrixes, and both fibers and proteins inhibit the incorporation of carotenoids into the micelles [60, 72]. While heating during cooking can degrade most nutrients in foods, such a treatment increases the bioavailability of certain nutrients, such as lycopene [73]. Therefore, understanding factors influencing bioaccessibility and bioavailability of carotenoids is crucial to achieving their ultimate bioefficacy.

## 5. Encapsulation

Nutrient bioavailability precedes its bioactivity at target tissues. In order to obtain the maximum bioefficacy of any given nutrients whose bioaccessibility and bioavailability are not satisfactory, a number of strategies are sought for their improvements. For example, encapsulation with food grade or related Generally Recognized As Safe (GRAS) materials has emerged as a novel strategy to improve the bioavailability and bioactivity of phytonutrients, including carotenoids. This encapsulation technology can include, but not limited to, microemulsions, matrix systems, solid dispersions, reassembled proteins, cross-linked polysaccharides, and liposomes [74–81]. The encapsulation, such as liposomes and emulsions, can stabilize carotenoids from possible degradation in the harsh gastrointestinal environment [82]. Nanoencapsulation is defined as a technology involving the formation of active loaded particles with diameters ranging from 1 to 1000 nm [83]. Particularly, polymeric nanoencapsulation has been adopted as one of preferred methods due to its higher loading capacity and better stability [84–86] and has been proven effective to augment bioavailability of carotenoids. For example, in a feeding study with male Swiss albino mice, Arunkumar et al. [87] reported that lutein nanoencapsulated by chitosan triphosphate was accumulated in a larger concentration in plasma, liver, and eyes as compared to the control. Furthermore, using an *in vitro* Caco-2 cell model, Yi et al. [88] found that solid lipid nanoentrapment significantly improved cellular uptake of *β*-carotene. Vishwanathan et al. [89] found in a small clinical trial that lutein supplemented in a stable hydrophilic nanoemulsion was 1.3-fold more bioavailable as evidenced in its serum status compared to lutein delivered in a pill. Thus, encapsulation can be a promising technology to enhance carotenoid bioaccessibility and bioavailability and to navigate precise delivery to target tissues such as eyes, brain, or/and skin for the maximum health benefits. However, clinical data supporting their applications remain largely lacking.

## 6. Safety and Efficacy of Carotenoids

It is well known that an excess of retinoids induces teratogenic effects [90, 91] and affects xenobiotic metabolism [92]. Although *β*-carotene is not teratogenic [9], high doses of *β*-carotene and vitamin E can be prooxidant and toxic [93, 94] and increase cancer risk. In particular, despite that high intake of *β*-carotene reduces the risk of many cancers (Table 3), the effect on breast cancer risk depends on estrogen receptor (ER) and progesterone receptor (PR) statuses [95] (Table 3). In general, the relationships between carotenoids and cancer risk depend on type of carotenoids and site of cancer, but the supplementation never confirms the suggestions from intake data (Table 3). Moreover, the increased risk of lung cancer after *β*-carotene supplementation had been reported in smokers and people drinking ≥11 g ethanol/d (ATBC study) [7]. The ATBC (20 mg/d) and CARET (30 mg/d) studies also showed increased risk for intracerebral hemorrhage [96], cardiovascular diseases [97, 98], and hyperlipidemia (in asbestos-exposed subjects) [98]. On the contrary, lycopene supplementation decreased LDL cholesterol [99] and blood pressure [100], at doses of ≥25 and > 12 mg/d, respectively, and lycopene has been suggested for preventing the toxic effects of antineoplastic drugs [101].

The overall mortality increased after *β*-carotene supplementation [102–104] at a dose of >9.6 mg/d [104]. On the contrary, for non-provitamin A carotenoids, an Observed Safe Level (OSL) of 20 mg/d for lutein and 75 mg/d for lycopene [13] has been suggested and an acceptable daily intake (ADI) of 53 mg/d has been proposed for zeaxanthin [105]. The positive effect of lutein and zeaxanthin on age-related macular degeneration is well known [106].

In the ATBC study, an induction of cytochrome P450 enzymes (CYP450) in male smokers supplemented with *β*-carotene has been reported [10]. Since CYP450 is the primary metabolizer of xenobiotics in humans, interactions between medication use and dietary supplements can occur. In this context, *β*-carotene supplementation (25,000 IU twice daily, 28 days) did not affect pharmacokinetics of nelfinavir and its active metabolite M8 in HIV-1-infected individuals [107], whereas a mixed supplement (400 IU/d of vitamin E, 500 mg/d of vitamin C, and 6 mg/d of *β*-carotene twice daily, 6 months) decreased cyclosporine A in renal transplant recipients [108]. Therefore, potential nutraceutical-drug interactions must be evaluated on the basis of the pharmacokinetics. Furthermore, interactions between alcohol and RA precursors are well documented and the combination of *β*-carotene with ethanol results in hepatotoxicity [109].

In particular, competitive inhibition of ADH could account for this adversity [110] and for the less adverse effects of non-provitamin A carotenoids (Table 3 and Table 4).

In the CARET study, *β*-carotene increased from 17 to 210 *μ*g/dL after 4 months of supplementation [111], whereas circulating lycopene concentrations between 2.17 and 85  *μ*g/dL were inversely associated with prostate cancer risk [112]. It shall be noted that such an association did not exist at concentrations greater than 85  *μ*g/dL [112]. It has been reported that circulating lycopene, rather than dietary lycopene, decreases stroke risk [113]. In this context, dietary guidance should consider upper limits for food-derived bioactive substances [114]. Also, efficacy should be determined in order to establish a therapeutic index of non-nutrient phytochemicals in foods and beverages [115].

## 7. Carotenoids and the Immune System

It is widely recognized that vitamin A deficiency decreases both humoral and cellular immune responses [16, 139] and that RA regulates innate immune response [140]. Vitamin A deficiency was associated with incidence of tuberculosis in human immunodeficiency virus- (HIV-) negative subjects [141] and in HIV-infected patients after antiretroviral therapy [142]. In addition, carotenoid concentrations were lower in tuberculosis cases before antiretroviral therapy [142]. However, in the ATBC study, *β*-carotene (20 mg/d) increased the risk of pneumonia in those who had initiated smoking at 21 years or later age [143] and the incidence of common cold in people undertaking strenuous exercise [144]. On the other hand, vitamins (vitamin C 120 mg, *β*-carotene 6 mg, and *α*-tocopherol 15 mg) with zinc (20 mg) and selenium (100 *μ*g) decreased the infectious events in elderly subjects [145]. However, low levels of vitamin A and carotenoids are associated not only with immunodeficiency but also with inflammation and autoimmunity and both systemic and GALT immune dysfunctions [18]. Patients with rheumatoid arthritis [146, 147], systemic lupus erythematosus [146], celiac disease [148], and/or Crohn's disease [149] had lower serum concentrations of carotenoids [149], *β*-carotene [146, 147], and/or retinol [146, 148]. Concerning non-provitamin A carotenoids, in the Third National Health and Nutrition Examination Survey (NHANES III), high serum lycopene concentrations were associated with lower mortality in patients with systemic lupus erythematosus [150].

Despite the potential concerted modulation of redox and inflammatory status, in a review of studies that investigated the effect of supplementation with antioxidant-rich foods or nutraceuticals on combined markers of redox and inflammatory status in humans, overall improvement in both markers of redox and inflammatory status was observed only in 27 studies of the 88 studies analyzed and only 28.6% (2/7) of the interventions with carrot, tomato, or lycopene-derived tomato (Lyc-O-mato) improved at least one marker of redox or inflammatory status [151]. Some serum inflammatory cytokines, such as tumor necrosis factor- (TNF-) *α* and interleukin- (IL-) 6, are also called adipomyokines [152] and are not specific markers of immune function, whereas their ex vivo production from peripheral blood mononuclear cells can be an index of immune response.

Table 5 describes major findings of human intervention studies [153–173] that investigated the effect of *β*-carotene, lycopene, mixed supplements, or carotenoid-rich juices and diet (fruits/vegetables) on immune function assays, including the *in vivo* test of cell-mediated immune response delayed-type hypersensitivity (DTH) and/or ex vivo assays of innate (i.e., natural killer (NK) activity and oxidative burst) and adaptive immunity (i.e., lymphocyte proliferation and cytokine production).

Increased levels of *β*-carotene [155, 156, 158–160, 163–165, 167–169, 172], lycopene [159, 161, 162, 167–169, 172], and lutein [167, 168] as well as of antioxidant vitamins (vitamin E and/or C) in the case of mixed supplements (Table 5) were found in response to treatment. Furthermore, increases in plasma carotenoid from 2.03 to 3.05 *μ*M were reported after 8 weeks of a consumption of 8 servings/d of vegetables and fruits, including carrots, green beans, peas, broccoli, zucchini, tomatoes, kohlrabi, Brussels sprouts, red cabbage, cauliflower, spinach, lettuce, radishes, cucumbers, fennel, apples, pears, kiwis, bananas, peaches, nectarines, cherries, strawberries, and red currants [173].

*β*-Carotene inhibited the ultraviolet light- (UV-) induced immunosuppression, evaluated with a DHT test in both healthy and elderly subjects, whereas contrasting results were reported on DHT when lycopene, *β*-carotene, or mixed supplements were used without UV irradiation (Table 5).

Data from ex vivo markers of adaptive immunity do not support an effect of lymphocytes' proliferation, whereas results concerning cytokine production are of difficult interpretation due to the differences in the dosage and duration of carotenoid supplementation and the use of carotenoid depletion periods (Table 5). In a longitudinal study of four periods, each lasting 2 weeks (weeks 1–2: low-carotenoid period; weeks 3–4: 330 mL tomato juice; weeks 5–6: 330 mL carrot juice; weeks 7–8 : 10 g dried spinach powder), tomato juice consumption increased IL-2 and IL-4 secretion compared with that at the end of the depletion period, whereas no effects were observed after carrot juice and spinach powder [170] (Table 5).

The same group [169] observed, in a crossover design, that ex vivo IL-2 production increased after carrot juice only in the arm depletion-carrot juice-depletion-tomato juice. TNF-*α* increased after the first supplementation (both juices) but only with carrot juice after the second supplementation [169]. Moreover, IL-2 further increased after supplementation and lymphocyte proliferation increased in both groups after the end of the first juice supplementation period despite that it did not change after carrot or tomato juice consumption compared with that at the end of the first low-carotenoid period [170]. Authors reported that this immunomodulation could not be explained by changes in the plasma carotenoid concentrations [170] and that provitamin A effect can be excluded because plasma retinol levels did not change after juice supplementation.

Concerning innate immunity, conflicting results were reported for oxidative burst-induced reactive oxygen species (ROS) production, whereas NK activity resulted to be increased in the majority of the studies (Table 5). However, the maximal increase in NK activity has been observed 1 week after juice supplementations had been stopped and the increase in NK cell activity is not associated to increase in NK percentage [157].

**Figure 1:**
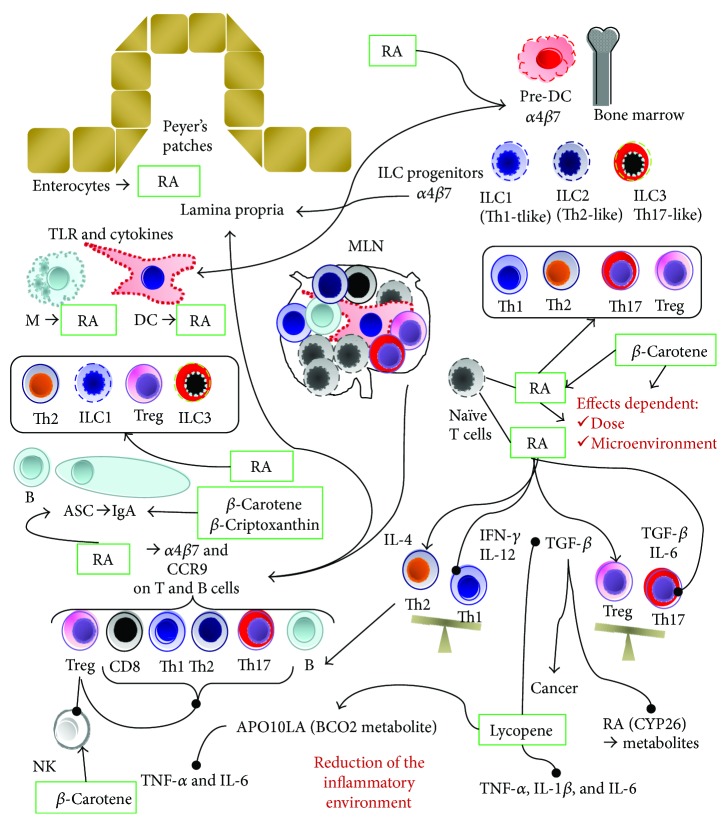
Immunomodulatory effects of carotenoids and retinoic acid. *α*4*β*7: *α*4*β*7 integrin; APO10LA: Apo-10′-lycopenoic acid; ASC: antibody-secreting cells; BCO2: *β*-carotene 9′,10′ oxygenase-2; CCR9: C-C chemokine receptor 9; CYP26: cytochrome P450 26; DC: dendritic cells; IFN: interferon; Ig: immunoglobulin; IL: interleukin; ILC: innate lymphoid cells; M: macrophages; MLN: mesenteric lymph nodes; NK: natural killer; RA: retinoic acid; TGF: transforming growth factor; Th: T helper; TLR: Toll-like receptor; TNF: tumor necrosis factor; Treg: regulatory T. →: homing and improvement; −•: inhibition.

Accordingly, results on lymphocyte subsets are conflicting. Despite that in older subjects *β*-carotene (30 mg/d, 2 months) increased plasma *β*-carotene and the percentage of NK, without affecting plasma retinol [174], many studies did not observe any effect on lymphocyte subsets after *β*-carotene supplementation [153, 158, 159, 165–167, 172, 173, 175]. Moreover, in a randomized controlled trial (RCT), *β*-carotene (30 mg/d) supplementation for 3 months in subjects with colonic polyps or colon cancers increased CD4 count only in cancer patients who had a lower percentage of CD4 than in patients with polyps and in controls [176]. On the other hand, *β*-carotene (60 mg/d) increased CD4^+^ cell counts only in patients with AIDS who have greater than 10 cells/microliters [177]. In HIV patients, *β*-carotene (60 mg/d, 3 months) increased NK, but not CD4 [178]. On the contrary, others reported that in HIV patients, *β*-carotene (60 mg/d orally three times daily and at 1 month and 3 months) did not change T cell subsets and NK, despite the increase in serum *β*-carotene [175]. Contrasting results came from supplementation with *β*-carotene in doses ranging from 60 mg/d to 180 mg/d on CD4 count in HIV patients [175, 177, 179–183], and data from a recent meta-analysis does not support *β*-carotene supplementation for increased CD4 cell count in patients with HIV [184]. However, GALT resulted to be depleted of CD4 also after restoration of blood CD4 by combined antiretroviral therapy (cART) [185]. In particular, it has been reported that HIV patients had defective gut homing of C-C chemokine receptor 9 (CCR9) and gut-homing *β*7 integrin on T helper cells producing IL-17 (Th17) [185]. In this context, it is well known that RA induces the gut-homing molecules *α*4*β*7 integrin and CCR9 in B and T (CD4 and CD8) cells [2, 3, 139] (Figure 1). RA can also induce *α*4*β*7 integrin and CCR9 on type 1 and 3 innate lymphoid cells (ILCs), but does not lead to CCR9 expression on type 2 ILCs [3, 18]. In terms of cytokine production, ILC1, ILC2, and ILC3 cells are Th1-like, Th2-like, and Th17-like cells, respectively [186] (Figure 1). Although plasticity has been suggested between ILC2/ILC1 and between ILC3/ILC1, ILC2 has been involved in asthma, lung fibrosis, esophagitis, and atopic dermatitis; ILC1 in chronic obstructive pulmonary disease and Crohn's disease; and ILC3 in psoriasis and obesity-associated inflammation [187]. Furthermore, ILC1 and ILC3 induce the polarization of inflammatory macrophages M1 [139]. Therefore, innate immunity can affect local inflammation.

In addition to the enterocytes' production, RA is also produced by stromal cells in the lamina propria (LP) and mesenteric lymph nodes (MLN), as well as by dendritic cells (DC) and macrophages [3] in the GALT. DC are major RA producers in LP, Peyer's patch, and MLN [188] (Figure 1). Preclinical studies suggest that the expression of gut-homing molecules by DC precursors in marrow is regulated by RA [18] (Figure 1). These cells migrate in the gut and induce oral tolerance by inducing regulatory T cells (Treg) [18]. RA induces also RA-producing CCR7^+^ DC that migrate to the MLN and induce gut homing in T cells [18] (Figure 1). RA production by DC is regulated by many local signals. Microbial-derived signals, by Toll-like receptor (TLR) 2 and TLR5, as well as butyrate produced by commensal bacteria, induce ALDH expression in DC [3, 18]. Besides IL-4 from ILC2 and Th2 cells, transforming growth factor beta (TGF-*β*) may also induce ALDH expression [3, 18]. The effects of RA on Th subsets depend on the local microenvironment [2, 3, 139].

In physiological conditions, RA produced by DC inhibits the differentiation of naïve T cells to Th17 cells by blocking IL-6, IL-21, and IL-23 signaling in naïve T cells [3]. RA-primed DC induce the production of the anti-inflammatory cytokine IL-10 in Tregs [3], and RA itself promotes TGF-*β*-mediated Treg conversion of naïve T cells [2, 3] (Figure 1). TGF-*β* is also involved in IgA class switching [189], and RA induces the expression of *α*4*β*7-integrin and CCR9 on B cells and antibody-secreting cells (ASC) [2, 189] (Figure 1). Furthermore, DC-derived RA, plus IL-5, IL-6, or TLR signals, has a primary role in the polarization of B cells in favor of IgA-producing ASC, by inducing IgA class switching in B cells [3, 18, 139, 189], and it has been suggested that oral RA administration before vaccine can increase the secretion of IgA into gut secretions [91]. Concerning provitamin A carotenoids, some preclinical studies suggest an effect on humoral immunity (Figure 1). In mice, 50 mg/kg *β*-carotene for 21 d increased the concentrations of IgA and the numbers of ASC in the jejunum [190]. Also, *β*-cryptoxanthin (5–10 mg/kg, 14 and 21 d) in rabbit increased the blood CD4, IL-4, and humoral immunity (IgG, IgM, and IgA) [191].

During inflammation, IL-1 enhances an IL-6-induced shift of the Treg/Th17 balance towards Th17 cells [3], and RA promotes, in the presence of IL-15, the secretion of IL-12 and IL-23 by DC, inducing the IFN-*γ*-producing Th1 and Th17 cells, and enhances the IL-4-mediated induction of Th2 [3, 18, 140]. On the other hand, in deficiency state, there are marked increases of ILC2 cell proliferation and cytokine (IL-4, IL-5, IL-6, IL-9, and IL-13) production, and, at the same time, the proliferation and function of ILC3 subset are suppressed [139].

It has been also suggested that RA has a dose-dependent effect: at pharmacological or high doses (10 nM and higher), RA inhibits Th17 and Th1 cells and induces Treg, whereas at physiological low doses (1 nM), RA favors Th17 cell differentiation [3, 16] (Figure 1). Th17 is involved in Crohn's disease [192], and the anti-α4*β*7 integrin therapeutic antibody (vedolizumab) targets gut-homing Th17 [193]. Although a reduced Treg/Th17 balance is often associated with inflammatory bowel disease, rheumatoid arthritis, systemic lupus erythematosus, and multiple sclerosis, the potential role of vitamin A or RA treatments is controversial [3].

IL-6 has a primary role in Th17 induction (Figure 1), and a recent meta-analysis reported that tomato supplementation was associated with significant reductions in IL-6 [136]. In a study using an animal model of ulcerative colitis (dextran sulfate sodium), *β*-carotene decreased colon IL-6 (5, 10, and 20 mg/kg), TNF-*α* (10 and 20 mg/kg), and IL-17 (20 mg/kg) and reduced plasma lipopolysaccharide [194]. On the other hand, intragastric lycopene administration (5 mg/kg [195]; 1, 2, and 4 mg/kg [196]) reduced TNF-*α*, IL-1*β*, IL-6, and/or TGF-*β* in a rat model of Alzheimer's disease and inhibited the *β*-amyloid-induced upregulation of TLR4 in the choroid plexus [195]. The effect on TGF-*β* has implication also in cancer (Figure 1). Lycopene inhibited TGF-*β*-induced migration, invasion, and adhesion activity of human liver adenocarcinoma SK-Hep-1 cells (2.5 *μ*M) [197] and decreased TGF-*β*1 mRNA levels in fibroblasts [198]. On the contrary, the role of RA in cancer is controversial.

Despite that RA is required for the expansion of tumor-reactive CD8 T cells, the induction of the TGF-*β*-producing Treg may inhibit tumor immunosurveillance [188]. In this context, TGF-*β* reduced the expression of CYP26, inhibiting the breakdown of RA [3] (Figure 1). Therefore, non-provitamin A carotenoids could have anti-inflammatory properties without compromising cancer immunosurveillance and could not increase cancer risk as observed after *β*-carotene supplementation (Table 3). However, although the activity of *β*-carotene on immune function could be due to its conversion to vitamin A and RA [19], it has been suggested that apo-10′-lycopenoic acid (apo10LA), a BCO2 metabolite of lycopene, activates the RAR, reducing IL-6 and IL-1*β* [199]. In mice, APO10LA at 10 mg/kg diet for 24 weeks reduced diethylnitrosamine-initiated, high-fat diet- (HFD-) promoted hepatic tumorigenesis, lung tumor incidence, and hepatic TNF-*α* and IL-6 concentrations [200]. Data from BCO2-knockout (BCO2-KO) and wild-type mice suggest that IL-6 inhibition and chemoprevention could depend on BCO2 expression [201]. Therefore, the role of metabolites from non-provitamin A carotenoids deserves future investigation.

## 8. Conclusion

From the reviewed data, the total carotenoid intakes range from 5.42 to 15.44 mg/d (Table 2) and the suggested recommended intake range are 2–4.8 mg/d for *β*-carotene [34, 37], 10–20 mg/d for lutein, and 5.7–15 mg/d for lycopene [38]. Higher intakes from foods rather than supplementation with *β*-carotene have been associated with healthy effects (Table 3 and Table 4), whereas more promising results came from lycopene supplementations (Table 4). However, the majority of the available data came from epidemiological studies and meta-analysis that include few RCT (<15) [99, 100, 136], with a small sample size (<100), and no supplementation data on cancer risk is available. Therefore, large-scale intervention studies are warranted to substantiate the health effects of lycopene.

Despite the antioxidant activity of *β*-carotene, the major provitamin A carotenoid, its prooxidant activity in smokers and alcohol drinkers justifies its adverse effects in doses ranging from 20 mg/d to 30 mg/d [96–98, 143]. The overall mortality increased after *β*-carotene supplementation at doses >9.6 mg/d [104], and potential food/drug or supplements/alcohol interactions can be also taken into account due to competition for and/or induction of metabolism enzymes [10, 108–110].

On the contrary, non-provitamin A carotenoids could have a safer profile (20 mg/d for lutein, 75 mg/d for lycopene, and 53 mg/d for zeaxanthin) [13, 105] than *β*-carotene. The latter is converted to RA with immunomodulatory effects (Figure 1).

Human intervention studies that investigated the effects of carotenoids on immune function involve *β*-carotene, lycopene, or food sources and suggest that carotenoids affect immune function only after a depletion period and at doses (≥30 mg/d *β*-carotene and lycopene) (Table 5) higher than recommended intakes. Some effects, unrelated to carotenoids and retinol plasma levels, have been observed after the end of the supplementation period. Furthermore, results on lymphocyte subsets are conflicting. In this context, local production of RA can affect the GALT and lymphocyte gut homing. The effect of RA on T-helper subsets depends on local microenvironment and inflammatory status. In this context, although RA is the major active metabolite affecting the immune system, preclinical data suggest that lycopene metabolites derived from BCO2 can modulate immune function by reducing the inflammatory cytokine IL-6 (Figure 1). In this context, there is a growing interest in BCO2 metabolites [202] and it is well known that based on genetic polymorphisms of BCO1 it is possible to cluster subjects as strong responders or weak responders to carotenoids [203, 204]. BCO1 polymorphisms also affect non-provitamin A carotenoids, such as lutein [205, 206] and lycopene [206]. This body of evidence suggests that personalized nutrition/supplementation should be considered in the future.

On the other hand, preclinical studies suggest that the differential effect of RA and lycopene on TGF-*β* can account for the safer profile of lycopene in the context of cancer incidence (Figure 1).

However, on the light of the different effects of RA at physiological and pharmacological doses [3, 16] (Figure 1), more studies are needed in order to establish the therapeutic index for lycopene and caution must be taken to extrapolate preclinical data to clinical uses. Furthermore, the majority of human interventions report the effects of lycopene on immune function administering mixed supplements or tomato products with lycopene ranging from 15 to 47.1 mg (Table 5). These doses are near or over the higher value of the suggested recommended intake (5.7–15 mg/d) [38], raising a safety concern.

In conclusion, although lycopene supplementation for immune-regulation seems more promising than *β*-carotene, human studies with adequate power and duration are needed in order to confirm this hypothesis.

## Figures and Tables

**Table 1:** Carotenoid food/spice content.

Range mg/100 g	*α*-Carotene	*β*-Carotene	*β*-Cryptoxanthin	Lutein + zeaxanthin	Lycopene
20–50		Carrot, paprika, peppers red			Tomatoes

10–20	Carrot	Carrots, peppers red		Chard, chicory greens, kale, paprika, pepper, spinach, turnip greens,	Catsup, tomatoes

5–10	Peppers red, pumpkin, carrot juice	Acai berry drink, carrot juice, carrots, chili powder, kale, parsley, pumpkin, spinach, turnip greens	Pepper, red or cayenne paprika	Basil, parsley, radicchio, watercress	Guavas, tomato juice, tomato soup

1–5	Carrot, chili powder, pepper	Apricots, broccoli, cabbage Chinese, cherries, chicory greens, endive, lettuce (green and red leaf), melons, oregano, parsley, peas green, peppers green, plums, pumpkin, sweet potato, thyme, watercress	Chili powder, squash	Broccoli, brussels sprouts, carrot, fava, lettuce (green and red leaf), oregano, parsley, peas green, pistachio, pumpkin, thyme, tomatoes, zucchini	Grapefruit (pink and red), papayas, watermelon

From: United States Department of Agriculture Agricultural Research Service (USDA) Food Composition Databases (https://ndb.nal.usda.gov/ndb/).

**Table 2:** Comparison of carotenoid intake (mg/d) in adults reported in several countries.

Population (subjects)	α-Carotene	*β*-Carotene	*β*-Cryptoxanthin	Lutein + zeaxanthin	Lycopene	Dietary methods	Ref.
Australia, *N* = 3100	1.25/1.13 (m/w)	5.14/5.27 (m/w)	0.32/0.35 (m/w)	1.62/1.70 (m/w)	7.11/6.26 (m/w)	FFQ	[39]
Costa Rica, *N* = 459	0.45/0.73 (m/w)	3.41/4.67 (m/w)	0.38/0.55 (m/w)	2.41/2.89 (m/w)	5.45/5.77 (m/w)	FFQ and 7-day diary	[40]
France, *N* = 76	0.74	5.84	0.45	2.50	4.75	FFQ	[32]
France, *N* = 12,741	—	3.14/3.79 (m/w)	—	—	—	6-day food diary	[41]
Ireland, *N* = 828	—	—	—	1.60	—	166-item FFQ	[42]
Italy (INRAN-SCAI study), *N* = 2313	0.15/0.18 (m/w)	3.07/3.01 (m/w)	—	3.79/3.73 (m/w)	7.10/5.64 (m/w)	3-day food diary	[43, 44]
Japan JACC Study Group, *N* = 3095	—	2.11 (m)	—	—	—	35-item FFQ	[45]
Korea National Health and Nutrition Examination Survey, *N* = 24,377	0.56	3.62	0.55	2.300	2.22	1-day 24 h recall	[46]
Spain, *N* = 70	0.29	2.96	1.36	3.25	1.64	FFQ	[32]
Spain (EPIC cohort), *N* = 41,446	0.27	1.31	0.22	0.84	3.0	Dietary history questionnaire	[47]
Rep Ireland, *N* = 76	1.23	5.16	0.78	1.56	4.43	FFQ	[32]
The Netherlands, *N* = 75	0.68	4.35	0.97	2.01	4.86	FFQ	[32]
USA, *N* = 584	0.69/0.79 (m/w^∗^)	3.28/0.63 (m/w^∗^)	0.15/0.17 (m/w^∗^)	1.47/1.56 (m/w^∗^)	6.07/5.35 (m/w^∗^)	118-items FFQ	[48]
	0.98/0.91 (m/w^∗∗^)	4.09/3.82 (m/w^∗∗^)	0.16/0.13 (m/w^∗∗^)	2.88/2.25 (m/w^∗∗^)	5.79/4.64 (m/w^∗∗^)		
USA, *N* = 2787	0.78 (w)	4.40 (w)	0.18 (w)	0.30 (w)	6.34 (w)	FFQ	[49]
UK, *N* = 71	1.04	5.55	0.99	1.59	5.01	FFQ	[32]
UK (EPIC Norfolk cohort), *N* = 14,803	0.41/0.40 (m/w)	2.07/2.04 (m/w)	0.41/0.46 (m/w)	1.10/1.14 (m/w)	1.43/1.29 (m/w)	7-day diary	[50]

FFQ: food frequency questionnaire; m/w: men/women; JACC: Japan Collaborative Cohort; EPIC: European Prospective Investigation into Cancer and Nutrition. ^∗^Hispanics and ^∗∗^Non-Hispanics.

**Table 3:** Carotenoids and cancer risk.

	*β*-Carotene	*α*-Carotene	*β*-Cryptoxanthin	Lycopene	Lutein + zeaxanthin
High intake				Ovarian (postmenopausal) ↓ [116]	
High intake	Bladder ↓ [117]	Bladder ↓ [117]	Bladder ↓ [117]	Bladder ↔ [117]	Bladder ↔ [117]
Supplement	Bladder ↑ [118]				
High intake	Breast (ER+, ER+/PR+) ↑ [95]	Breast ↓ [95]	Breast ↓ [95]	Breast (ER−/PR+ or ER−/PR−) ↓ [95]	Breast (ER−/PR+ or ER−/PR−) ↓ [95]
	(ER−/PR+ or ER−/PR−) ↓ [95]				
Supplement	Gut (colorectal) ≈↑ [119]				
High intake	Gut (esophageal) ↓ [120]	Gut (esophageal) ↓ [120]	Gut (esophageal) ↓ [120]	Gut (esophageal) ↓ [120]	Gut (esophageal) ↓ [120]
High intake	Gut (gastric) ↓ [121, 122]	Gut (gastric) ↓ [121, 122]		Gut (gastric) ↔ [121, 123]	Gut (gastric) (lutein) ↔ [121]
Supplement	Gut (liver) ↔ [124]				
High intake	Gut (pancreatic) ↓ [125]	Gut (pancreatic) ↔ [125]	Gut (pancreatic) ↓ [125]	Gut (pancreatic) ≈↓ [125]	Gut (pancreatic) ↔ [125]
Supplement	Gut gastric ≈↑ [126]				
Supplement	Gut intestinal ↑ [126]				
High intake	Hodgkin lymphoma ↓ [127]	Hodgkin lymphoma ↓ [127]	Hodgkin lymphoma ↔ [127]	Hodgkin lymphoma ↔ [127]	Hodgkin lymphoma ↓ [127]
High intake	Lung ↓ [128]				
Supplement	Lung ↑ [7, 129]				
High intake	melanoma ≈↓ [130]				
Supplement	Oral ↔ [131]				
High intake	Oral ↓ [132]	Oral ↓ [132]	Oral ↓ [132]	Oral ↓ [132]	
High intake	Prostate ↔ [133]	Prostate ↓ [133]		Prostate ↓ [112, 133]	
Supplement	Prostate **↔** [134]				

≈ ns. increase or decrease; ↓: decrease; ↑: increase; ↔: no change; ER: estrogen receptor; PR: progesterone receptor.

**Table 4:** Effects of lycopene and *β*-carotene supplementation on cardiometabolic outcomes.

	Lycopene	Lutein	*β*-Carotene
Blood lipids	↓ Cholesterol [99]	↔ [135]	↑ Cholesterol and triglycerides (asbestos-exposed) [98]
	↔ Cholesterol [136]		
Diabetes/insulin resistance		↔ Insulin resistance [135]	↔ Type 2 diabetes [137]
Diabetic macrovascular disease			↔ [138]
Metabolic syndrome		↓ [135]	
Blood pressure	↓ [100, 136]	↔ [135]	
CVD and nonfatal myocardial infarction			↑ [97, 98]
Stroke	↓ [113]		
Intracerebral hemorrhage			↑ [96]
CV death			↑ [103]

↓: decrease; ↑: increase; ↔: no change; CVD: cardiovascular disease; CV: cardiovascular.

**Table 5:** Effects of carotenoid and carotenoid-rich food and beverages on test of immune function.

Subjects (study)	Treatment	Outcomes [ref.]
Healthy (RCT)	*β*-Carotene (15–120 mg), 4–7 wk	↔ lymphocyte proliferation [153], ROS production [154]
		↑ DTH (30 mg) versus control (↓ after UV exposure response only in the placebo group) [155]

Elderly (RCT)	*β*-Carotene (8.2, 30, 50, and 90 mg), 3–6 wk to 10–12 y	↑ DTH (30 mg) versus control (↓ after UV exposure response only in the placebo group) [156]
		↑ NK activity [157]
		↔ production of IL-12 and IFN-*γ* (50, 90 mg) [157]
		↔ DTH (50 and 90 mg), production of IL-2 [158], and lymphocyte proliferation [158, 159]

Smokers (RCT)	*β*-Carotene (40 mg), 4 and 6 wk	↓ ROS production [160]

Type 2 diabetes (RCT)	Lycopene (10 mg/d), 8 wk	↔ DHT [161, 162]

Elderly (RCT)	Lycopene (13.3 mg), 12 wk	↔ lymphocyte proliferation [159]

Elderly (RCT)	Mixed supplement	
	*β*-Carotene (0.75 mg), vitamin C (90 mg), and vitamin E (20 mg), 1 y	↑ DHT [163]
	*β*-Carotene (6 mg), vitamin C (120 mg), and vitamin E (15 mg), 1–2 y	↔ DHT [164], lymphocyte proliferation [165]

Healthy (RCT)	Mixed supplement	
	*β*-Carotene (12 mg), vitamin E (288 mg), and vitamin C (375 mg), 6 and 10 wk	↑ DTH [166]
		↔ lymphocyte proliferation, ROS production [166]
		↔ DHT [167]
	*β*-Carotene (30 mg), lycopene (15 mg), and lutein (9 mg), 5 wk	↓ IL-2 [167] and ROS [167, 168] production versus depletion (↑)

Healthy (RCT/longitudinal)	Carrot juice (330 mL, 21.6–27.1 mg *β*-carotene, and 13.1–15.7 mg *α*-carotene), 2 wk	↑ TNF-*α* versus depletion (arm carrot juice-tomato juice, arm tomato juice-carrot juice) [169]
		↑ IL-2 versus depletion (arm carrot juice-tomato juice) [169]
		↔ lymphocyte proliferation and IL-4 production [169, 170]
		↑ NK activity [169]

Healthy (longitudinal)	Dried spinach powder 10 g (11.3 mg lutein and 3.1 mg *β*-carotene), 2 wk	↔ lymphocyte proliferation, IL-2 and IL-4 production [170]

Healthy (RCT/longitudinal)	Tomato-based drink (Lyc-o-Mato) (5.7 mg lycopene, 1 mg *β*-carotene, and 1.8 mg *α*-tocopherol), 26 days Tomato juice (330 mL, 37.0–40 mg lycopene and 1.5 mg *β*-carotene), 2 wk	↓ TNF-*α* production [171]
		↔ IFN-*γ* production (versus baseline, ↑ in placebo versus baseline) [171]
		↔ lymphocyte proliferation [169, 170], IL-2 and IL-4 production [169]
		↑ TNF-*α* versus depletion (arm tomato juice-carrot juice) [169]
		↑ IL-2 and IL-4 production versus depletion (↑) [170],
		↑ NK activity [169]

Elderly (RCT)	Tomato juice (330 mL, 47.1 mg lycopene), 8 wk	↔ DTH, lymphocyte proliferation [172]
		↓ IL-2 production (versus baseline, ns versus water) [172]
		↑ activity of NK, IL-4, and TNF-*α* production (versus baseline, ns versus water) [172]

Healthy (RCT)	Vegetables and fruit: 2, 5, or 8 servings/d, 4 wk	↔ NK activity, IL-2, IL-12, IFN-*γ*, TNF-*α* production, lymphocyte proliferation [173]

↓: decrease; ↑: increase; ↔: no change; d: days; DTH: delayed-type hypersensitivity; IFN: interferon; IL: interleukin; mo: months; NK: natural killer cells; RCT: randomized controlled trials; TNF: tumor necrosis factor; UV: ultraviolet light; wk: weeks; y: years.
